# Measurement Invariance of the Depression Anxiety Stress Scales-21 Across Gender in a Sample of Chinese University Students

**DOI:** 10.3389/fpsyg.2018.02064

**Published:** 2018-10-31

**Authors:** Shan Lu, Shuqing Hu, Yuhuan Guan, Jing Xiao, Dan Cai, Zhihua Gao, Zhiqin Sang, Jie Wei, Xiaochi Zhang, Jürgen Margraf

**Affiliations:** ^1^College of Psychology, Capital Normal University, Beijing, China; ^2^Department of Psychology, Shanghai Normal University, Shanghai, China; ^3^College of Psychology, North China University of Science and Technology, Tangshan, China; ^4^Department of Sociology, Nanjing University, Nanjing, China; ^5^Department of Education, Guizhou University of Finance and Economics, Guiyang, China; ^6^Department of Clinical Psychology and Psychotherapy, Mental Health Research and Treatment Center, Ruhr-Universität Bochum, Bochum, Germany

**Keywords:** measurement invariance, DASS-21, gender difference, confirmatory factor analysis, Chinese

## Abstract

The Depression Anxiety Stress Scales-21 (DASS-21) has three 7-item subscales (depression, anxiety, and stress). The current study aims assess the gender-based measurement invariance of the DASS-21 questionnaire in a Chinese university student sample from five different cities. The sample was composed of 13208 participants (62.3% female, mean age of 19.7 years, and *SD* age = 1.8). Multi-group confirmatory factor analysis supported full measurement invariance for the three subscales. The findings support the measurement invariance of DASS-21 scores across gender. Future research on the DASS should include additional validation across ethnicities and testing of all versions of the DASS.

## Introduction

The increased prevalence of anxiety and mood disorders has become a widespread challenge. The World Health Organization [WHO], 2017 estimated that 3.6% of people worldwide had anxiety disorders and 4.4% had depressive disorder in 2015 (World Health Organization [WHO], 2017). Numerous psychiatric epidemiological studies have indicated that women are significantly more likely than men to develop anxiety disorders and depression throughout the lifespan (Bruce et al., 2005). The rate of anxiety is 4.6% among women and 2.6% among men; it is thus less common than depression, which is present in 5.1% of females and 3.6% of males worldwide (World Health Organization [WHO], 2017). In China, the prevalence of depression was 21% (Zong et al., 2010). Song et al. (2008) reported that the prevalence of depression among university students in Hong Kong was 17.6%, with a gender difference marked by a higher prevalence among females (21.3%) than among males (13.4%). Available data also indicate that gender differences exist in recall, interpretation, and self-reporting of afflict inventory items (e.g., “I couldn’t experience any positive feeling at all.”, “I found it difficult to relax.”) (Grant and Weissman, 2007), and that women have a greater tendency than men to provide more severe responses to self-report inventory items (Ritvo et al., 2008).

Psychologists have developed several instruments for the assessment of the severity of the core symptoms of depression, anxiety, and stress (Selby et al., 2009). Clark and Watson (1991) proposed a tripartite model of these symptoms: general distress or negative affect, which occurs in anxiety and depression; physiological hyperarousal, which is common in anxiety; and low levels of positive affect, which are specific to depression (Barlow et al., 1998). Hence, Lovibond and Lovibond (1995) developed the 42-item Depression, Anxiety, and Stress Scales (DASS-42), a self-report instrument with three dimensions. Unlike most similar scales, such as the Self-Rating Depression Scale (Zung, 1965) and the Self-Rating Anxiety Scale (Zung, 1971), which are self-administered 20-item surveys used to quantify depression and anxiety statuses separately, the DASS is a single instrument used for the combined assessment of depression, anxiety, and stress. The DASS-21 was developed from the original DASS-42 by selecting 7 of 14 items for each subscale with the highest loadings. Brown et al. (1997) verified that the three subscales measure the three dimensions, with the depression scale associated with low positive affect, the anxiety scale associated with physiological hyperarousability, and the stress scale associated with negative affect.

Since its development in 1995, the DASS has been used widely in multiple settings, in clinical and non-clinical samples (Crawford and Henry, 2003; Henry and Crawford, 2005; Page et al., 2007), in different countries (Taouk et al., 2001; Daza et al., 2002), and for different age groups (Gloster et al., 2008; Szabó, 2010; Osman et al., 2014). To date, the DASS has been translated into 42 languages (Crawford et al., 2011), which makes it widely accessible for practitioners and researchers. Zuo and Chang (2008) completed a direct translation of the DASS from the original English into the simplified Chinese character set. Chan et al. (2012) confirmed the psychometric properties of the Chinese version of the DASS for use in China.

However, a prerequisite for use of the same psychological scale score for different groups is cross-group measurement invariance (Reise et al., 1993). Other scales that measure depression and anxiety (e.g., Reynolds Adolescent Depression Scale, Beck Depression Inventory-II) have been proven to have reliable measurement invariance in multiple studies (Fonseca-Pedrero et al., 2010; Wu and Huang, 2014). Few studies have tested the measurement invariance of the DASS in terms of gender. Gomez et al. (2014) found that the ratings of DASS-21 have measurement and structural invariance across gender in a sample of the United States. But they treated the data of DASS-21 as continuous and using maximum likelihood estimation, it is appropriate for a 4-Likert scale and would like to keep treating the data correctly as categorical using WLSMV. And no similar work has been conducted for the Chinese version of the DASS or DASS-21. Psychometric properties need to be investigated within the new population to ensure that it functions like the original instrument (Scholten et al., 2017). In this study, we aim to extend previous research by verifying the measurement invariance of the DASS-21 across gender groups in Chinese university students using multigroup confirmatory factor analyses (CFA).

## Materials and Methods

### Participants and Procedure

Data for this study were taken from the Bochum Optimism and Mental Health research program, a cross-cultural effort to test the measurement invariance of the DASS-21. Our study included samples from five universities in China (Capital Normal University, Nanjing University, Shanghai Normal University, Hebei United University, and the Guizhou University School of Finance Economics). Collaborating departments of psychology at the universities collected the data. As all data were anonymized from the beginning of the research, no institutional board or ethics committee approval was required. At all universities, local laws granted officially inscribed university students of all ages the right to make decisions about study-related issues, including study participation.

The sample comprised 13,208 students (4985 men, 8223 women). The mean (standard deviation [SD]) ages of the sample was 19.7 (1.8), in which male and female participants were 19.9 (2.1) years and 19.6 (1.7) years, respectively. No data were missing for any participating student.

A written version of the DASS-21 was administered to all students. Participants were informed before the test that their responses would be anonymous and utilized only for the study.

### Measure

The DASS-21 is a self-report measure of negative affect with three 7-item subscales (depression, anxiety, and stress). The psychometric properties of the short version of the DASS are well established (Lovibond and Lovibond, 1995). Item examples include “I experienced trembling in the hands” (Anxiety), “I felt that I had nothing to look forward to” (Depression), and “I found it hard to wind down” (Stress) (Mellor et al., 2014). Responses are structured by a 4-point Likert scale ranging from 0 (“does not apply to me at all”) to 3 (“applies to me very much or most of the time”), with higher scores indicating more negative experience in the past week. Scores for each subscale are obtained by summing the responses to the component items.

In the current study, the Chinese version (simplified characters) of the DASS-21 was retrieved from the DASS website (Lovibond, 2015). The alpha coefficients for the reliability of the depression, anxiety, and stress scales in the entire group were 0.82,0.82, and 0.79, respectively.

### Data Analysis

All statistical analyses were conducted using SPSS 19.0 (Statistical Package for the Social Sciences[SPSS] 2010) and M*plus* (Muthén and Muthén, 1998). We employed weighted least squares with mean and variance adjusted (WLSMV) because of the 4-point nature of the DASS-21 response scale. WLSMV is recommended to estimate thresholds when fewer than five response categories are used (Beauducel and Herzberg, 2006). Delta parameterization was used because it is also recommended for ordered-categorical data (Muthén and Muthén, 2017)

We sought to establish well-fitting baseline models for the male and female samples separately (Byrne, 2008; Hirschfeld and Von Brachel, 2014), based on the established three-factor structure of the DASS-21 (Lovibond and Lovibond, 1995; Antony et al., 1998; Taouk et al., 2001; Daza et al., 2002; Crawford et al., 2011) We did the exploratory factor analysis (EFA) to test the three-factor structure. As Chan et al. (2012) mentioned, the approach with EFA for DASS was based on that used by Lovibond and Lovibond (1995). A principal component analysis (PCA), which specifies three components with oblique rotation (Oblimin; delta = 0), was conducted to demonstrate if the three correlated dimensions of the test are possible. Oblique rotation method is employed when the obtained factors are related to one another.

We used single-group confirmatory factor analysis (CFA) to verify good fit across gender before testing measurement invariance (Van de Schoot et al., 2012).

Model fit was estimated using the χ^2^ statistic, the comparative fit index (CFI), and the root mean square error of approximation (RMSEA). All indices were based on robust values corrected in accordance with the WLSMV estimator. Following Hu and Bentler (1999), we considered CFI > 0.90 to indicate “adequate” model fit. We considered RMSEA values <0.06 to indicate “good” model fit, values of 0.07–0.08 to indicate moderate fit, and those of 0.08–0.10 to indicate marginal fit (Hu and Bentler, 1999).

### Measurement Invariance

Multiple-group CFA was conducted to assess measurement invariance of the DASS-21 across gender. First, we estimated a configural invariance model (M1). Metric (weak) invariance was then tested in the nested model 2 (M2), in which factor loadings were equal across gender, and scalar (strong) invariance was tested in the nested model 3 (M3), in which the loadings and thresholds were constrained to be equal across genders (Van de Schoot et al., 2012). We considered metric and/or scalar invariance to be indicated when the corresponding model(s) (M2 and/or M3) fit the data at least as well as did M1. Support for both forms of invariance was considered to indicate meaningful comparability of the DASS-21 across genders, or cross-group equality (Millsap, 1998). For model comparisons, the chi-square difference tests were conducted using the DIFTEST option in M*plus*. As the χ^2^ test is known to be oversensitive in the assessment of invariance in large samples (*N* > 300) (Chen, 2007), the changes in CFI and RMSEA between the comparison and nested models were used for this assessment. Following Cheung and Rensvold (2002) and Chen (2007), we considered measurement invariance to be indicated by ΔCFI < 0.010 and ΔRMSEA < 0.015.

## Results

### Descriptive Statistics

The means and SDs for DASS-21 item scores ranged from 0.16 to 0.78 and from 0.46 to 0.87, respectively. The mean (SD) scores for the DASS-21 depression, anxiety, and stress subscales were 2.17 (3.26), 2.48 (2.59), and 3.69 (3.67), individually. On the basis of the DASS manual, these scores are within the normal ranges (Lovibond and Lovibond, 1995). On the whole, the sample examined in this study had normal levels of depression, anxiety, and stress. Cronbach’s alpha coefficients for depression anxiety and stress scales between male and female are 0.81 (0.75), 0.76 (0.72), and 0.80 (0.75).

### Exploratory Factor Analysis

**FIGURE 1:**
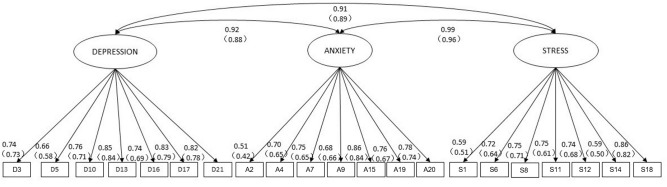
Baseline model of theDASS-21. The standardized factor loadings and factor covariance for the three-factor model for male (female). The residuals are not shown in the figure. D, depression; A, anxiety; S, stress.

The principle components solution accounted for 46.1% of the item variance. The Initial Eigenvalues of three components is all greater than 1. And the resulting structure (pattern matrix) of EFA is as, respectively, Stress, Depression, and Anxiety, but item loadings were not in all cases as they should be. The pattern matrix between the genders is almost the same. Components 1 and 2 correlated −0.42, 2, and 3 correlated −0.39, and 1 and 3 correlated 0.50. Three correlated components were identifiable, so we add the correlation to three factors in the CFA as shown in Figure 1.

### Model Fit for Single Groups

The results of the confirmatory factor analysis of the assumed three-factor model in each gender suggest that the model is appropriate across gender. The standardized factor loadings and factor covariance of each scale by gender see Figure 1. For the female sample, the results were as follows: χ^2^ = 3400.268, degrees of freedom = 186, *p* < 0.001; CFI = 0.960, and RMSEA = 0.046 (90% confidence interval [CI], 0.045–0.047). The values for the male sample were: χ^2^ = 2365.825, degrees of freedom = 186, *p* < 0.001; CFI = 0.965, and RMSEA = 0.048 (90% CI, 0.047–0.050). For both groups, the RMSEA values indicated good fit and the CFI values indicated acceptable fit.

### Multiple-Group CFA of Invariance Across Gender

The results for measurement invariance are displayed in Table 1.

**Table 1:** Results of tests for invariance across genders.

	Model fit				Model difference				
Model	*χ*^2^	df	RMSEA (90% CI)	CFI	ΔM	*Δχ*^2^	Δdf	ΔRMSEA	ΔCFI
M1	5795.513^∗^	372	0.047(0.046–0.048)	0.961	–	–	–	–	–
M2	5822.847^∗^	390	0.046(0.045–0.047)	0.961	M2 vs. M1	161.106^∗^	18	0.001	0.000
M3	5063.079^∗^	429	0.040(0.039–0.041)	0.967	M3 vs. M2	114.260^∗^	39	0.006	0.006

*RMSEA, root mean square error of approximation; CI, confidence interval; CFI, comparative fit index. M1, configural invariance; M2, metric invariance; M3, scalar invariance. ^∗^*p* < 0.01.*

#### Configural Invariance Across Gender

The three-factor configural invariance model fit the data very well (RMSEA = 0.047 [90% CI,0.046–0.048], CFI = 0.961). Moreover, all factor loadings were significant (*p* < 0.05) and ranged from 0.326 to 0.901. Thus, the metric invariance model was tested by constraining the factor loadings across gender.

#### Metric Invariance Across Gender

A constrained metric invariance model showed an acceptable fit (RMSEA = 0.046 [90% CI, 0.045–0.047], CFI = 0.961). Moreover, ΔCFI and ΔRMSEA were within recommended guidelines, supporting metric invariance. Given this support, we proceeded to test for scalar invariance.

#### Scalar Invariance

The scalar invariance model (M3) fit the data soundly well (RMSEA = 0.040 [90% CI, 0.039–0.0341], CFI = 0.967). In addition, the ΔCFI and ΔRMSEA values supported the scalar invariance model, which fit as well as the configural model (Table 1, M3).

## Discussion

The results of this study support the gender-based measurement invariance of the DASS-21 in a Chinese sample. Single-group CFA and configural invariance results supported the three-factor structure of the DASS-21 for both genders, which means that the DASS-21 measured the same constructs among male and female Chinese college students. Moreover, the support for metric invariance suggests that the DASS-21 items measure depression, anxiety, and stress in the same manner in both genders. Furthermore, the acceptable scalar invariance between males and females indicated that the intercepts of DASS-21 items were equal. These results indicate that the comparison of DASS-21 scores across genders is meaningful in Chinese samples. Therefore the same scale scores from these two groups reflect the same level of depression, anxiety, or stress. So we don’t need to use different normative scores for men and women. Although, as mentioned in the introduction, there is a significant difference in the prevalence of depression and anxiety symptom among men and women, it does not affect the use of DASS-21 in China. Our results are in agreement with the findings of Gomez et al. (2014) who demonstrated that DASS-21 is gender-invariant using the Australian adult sample (mean age 47.37). Our research on Chinese college students also reached the same conclusion, indicating that DASS-21 is not affected by gender in its application. The findings of this study contribute to the continuing validation of the DASS-21 in non-clinical settings, and we believe that they provide additional support for the use of the DASS-21, extend previous studies in applied and research settings.

This study has some limitations that should be addressed in future studies. First, the sample contained college students from a variety of ethno-racial groups. Fifty-six ethnic groups are represented in the population of China. With the integration of multi-ethnic cultures in China, the differences between the various ethnic groups have narrowed, so we did not take possible ethnic differences into account when we originally designed the experiment. Future research on the DASS should thus include additional validation across ethnicities. Second, the study sample was mainly composed of normal university students. In China, normal schools are educational institutions for teacher training, and they are attended by more female than male students. Given the obvious difference in the numbers of female and male students in such universities, whether the findings can be generalized to other universities is uncertain. Not only that, but our sample is college students, who are of similar age and education level, which may make the sample not highly representative. Future research can also expand the range of the sample and collect more people of different ages and levels of education. Third, because we examined measurement invariance using the short version of the DASS, we should notice that the findings may be specific to this version. We anticipate that the invariance of all versions of the DASS will be tested in future studies. Future studies of the measurement equivalence and DASS measures across gender would be useful.

## Ethics Statement

Data from students of universities in China were gathered by the collaborating Departments of Psychology specifically for the BOOM research program by Ruhr-University. The study is in total was approved by the ethics committee of the Faculty of Psychology at Ruhr-University on May 12, 2011. Approval to administer the questionnaires was granted by the Faculty of Psychology at Ruhr-University Bochum on May 12, 2011 and renewed on October 2012. The approvals for the German site were communicated to the participating Chinese Universities who acknowledged these approvals. Chinese laws grant officially inscribed University students of all ages the rights to decide for themselves about study-related issues including participation in studies.

## Author Contributions

JM and XZ contributed to the study conception and design of the study. DC, ZG, ZS, JW, SL, and XZ organized the database. YG and JX conducted the data analysis and drafted the manuscript. SH and SL edited and provided critical revisions to the manuscript. XZ offered assistance with data analysis and interpretation. All authors contributed to manuscript revision, read and approved the submitted version.

## Conflict of Interest Statement

The authors declare that the research was conducted in the absence of any commercial or financial relationships that could be construed as a potential conflict of interest.
